# Prediction of progression of damage to articular cartilage 2 years after anterior cruciate ligament reconstruction: use of aggrecan and type II collagen biomarkers in a retrospective observational study

**DOI:** 10.1186/s13075-017-1471-1

**Published:** 2017-12-06

**Authors:** Yasumori Sobue, Toshihisa Kojima, Kazutoshi Kurokouchi, Shigeo Takahashi, Hiroaki Yoshida, Robin Poole, Naoki Ishiguro

**Affiliations:** 1 Department of Orthopedic Surgery, Nagoya University School of Medicine, 65 Tsurumai, Showa, Nagoya 466-8550 Japan; 2 Orthopedic Surgery, Mitsubishi Nagoya Hospital, 7-8 Sotodoi, Atsuta, Nagoya 456-0013 Japan; 3 Orthopedic Surgery, Kamiiida Daiichi General Hospital, 2-70 Kamiiidakita, Kita, Nagoya 462-0802 Japan; 4 Division of Orthopaedics, Department of Surgery, McGill University, Montreal, QC Canada

**Keywords:** Cartilage, Biomarker, Anterior cruciate ligament injury, Keratan sulfate, Chondroitin sulfate, Arthroscopy

## Abstract

**Background:**

We aimed to determine whether synovial fluid (SF) biomarkers can predict the progression of articular cartilage damage as determined by arthroscopic evaluation during and after anterior cruciate ligament (ACL) reconstruction.

**Methods:**

Arthroscopic assessment of articular cartilage damage was performed twice in 62 patients, first during ACL reconstruction and then approximately 2 years later during implant removal for ligament fixation. SF levels of the collagenase-generated cleavage neoepitope of type II collagen (C2C) and proteoglycan glycosaminoglycans keratan sulfate (KS), chondroitin-4-sulfate (Δdi-C4S), and chondroitin-6-sulfate (Δdi-C6S) were measured at ACL reconstruction. Associations between baseline biomarker levels and subsequent progression of cartilage damage were determined using receiver operating characteristic analysis and multivariable logistic regression analysis.

**Results:**

No radiographic changes were observed in any of the patients. Progression of high-grade cartilage damage, observed arthroscopically, was negatively correlated with levels of Δdi-C6S and KS, as well as the ratio of Δdi-C6S to Δdi-C4S (C6S/C4S). Logistic regression analysis revealed significant associations of Δdi-C6S (cut-off: 55.7 nmol/ml, odds ratio (OR) 0.231, 95% confidence interval (CI) 0.061–0.879), KS (cut-off: 10.6 μg/ml, OR 0.114, 95% CI 0.024–0.529), and C6S/C4S ratio (cut-off: 4.6, OR 0.060, 95% CI 0.005–0.737) with the progression of high-grade cartilage damage after adjusting for age, the duration from injury to first surgery, sex, and the number of high-grade lesions (grades III and IV) at baseline.

**Conclusions:**

The progression of high-grade cartilage damage was significantly associated with baseline levels of proteoglycan glycosaminoglycan biomarkers; namely, Δdi-C6S, KS, and C6S/C4S ratio.

**Electronic supplementary material:**

The online version of this article (doi:10.1186/s13075-017-1471-1) contains supplementary material, which is available to authorized users.

## Background

Destruction of the articular cartilage matrix is one characteristic of osteoarthritis (OA), which can be associated with intolerable pain and difficulties in daily life such as walking, going up and down stairs, and other movements accompanying flexion and extension of the knee. Progression of cartilage damage cannot be prevented once radiographic changes appear. Therefore, detection of early cartilage damage is necessary in order to prevent OA progression.

The articular cartilage matrix is mainly composed of type II collagen and proteoglycans such as aggrecan. Collagen fibrils provide tensile strength to maintain tissue integrity, whereas aggrecan, interwoven with collagen fibrils, contributes to compressive stiffness [1]. Excessive cleavage of type II collagen by collagenases is thought to represent a critical step leading to the destruction of the cartilage matrix. In fact, the collagenase-generated cleavage neoepitope of type II collagen (C2C) is a useful biomarker that reflects the cleavage of type II collagen in patients suffering from anterior cruciate ligament (ACL) injury [2, 3] or OA [4, 5]. Aggrecan is the major cartilage proteoglycan. It consists of a core protein to which glycosaminoglycan side chains of keratan sulfate (KS) and chondroitin sulfate (CS) are attached [6, 7]. Chondroitin-6-sulfate (Δdi-C6S) and KS are most abundantly found in normal adult articular cartilage, where they are replaced with chondroitin-4-sulfate (Δdi-C4S) of newly synthesized proteoglycan as cartilage degradation progresses [8–10].

We recently reported that synovial fluid (SF) C2C and KS biomarkers are associated with the degree of cartilage damage based on arthroscopic evaluation in patients with ACL injury [1]. The number of high-grade cartilage lesions was associated with levels of C2C and KS. Moreover, our findings suggested that a combination of higher C2C and lower KS may offer a greater ability to identify patients with pre-radiographic, early high-grade cartilage damage compared to a single clinical or biomarker parameter.

Given the importance of longitudinal studies in clarifying the value of biomarkers to detect progression of cartilage damage before radiographic changes appear, this study aimed to investigate whether the progression of cartilage damage after ACL reconstruction was related to SF biomarkers measured at the time of ACL reconstruction, and whether they could predict progression of cartilage damage based on longitudinal arthroscopic evaluation.

## Methods

### Patients

Among 108 patients with ACL injury who participated in our previous study [1], 92 and 16 underwent ACL reconstruction between January 2001 and March 2003 at Mitsubishi Nagoya Hospital (Nagoya, Japan) and Kasugai Orthopedic Hospital (Kasugai, Japan) respectively. Reconstruction of the ACL was performed using the hamstring tendon with femoral fixation by the EndoButton CL device (Smith & Nephew Endoscopy, Andover, MA, USA) and tibial fixation by staples.

Of the 108 patients, 62 were followed and underwent two arthroscopic evaluations, one during ACL reconstruction (baseline) and another upon implant (staples) removal for tibial fixation of the reconstructed ligament approximately 2 years post-operatively (follow-up). All 62 patients who participated in this study were cases of Mitsubishi Nagoya Hospital. Because the staples are felt subcutaneously and cause pain or a feeling of alien substance, they are removed in most cases. Reasons for patient drop-out from our follow-up study were unclear, but there were no significant differences in patient baseline characteristics except for KS levels between the 62 patients in this study and the other remaining 46 patients (Additional file 1).

The study was approved by the Ethics Committee of Nagoya University School of Medicine (2017-0140). This is retrospective research including the information on synovial fluid obtained from the previous study. Therefore, we disclosed the study information at the site of the related facilities instead of using a consent form according to committee procedure.

### Arthroscopic evaluations of articular cartilage and menisci

**Fig. 1:**
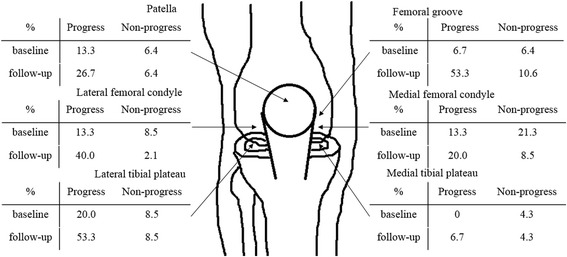
Proportions (%) of high-grade cartilage damage in progression and non-progression groups at baseline and follow-up. Arthroscopic grading was performed on each of six articular sites (*as shown*) according to the Outerbridge scoring system. High-grade cartilage damage was defined as Outerbridge grades III and IV. Baseline, ACL reconstruction; follow-up, implant removal; progression group, patients with an increase (between baseline and follow-up) in the number of high-grade cartilage lesions (Outerbridge grades III and IV) by one score in any of the six compartments; non-progression group, patients with no increase in the number of high-grade cartilage lesions

As was done in our earlier study [1], articular cartilage damage was evaluated at six sites (Fig. 1), including the patella, femoral groove, lateral femoral condyle, medial femoral condyle, lateral tibial plateau, and medial tibial plateau. We again used the Outerbridge grading system [11], which assesses both the depth and size of macroscopic chondropathic changes involving the articular cartilage surface in a range of grade 0 (normal) to IV. Grade I is characterized by softening and swelling of the cartilage. Grade II reflects the presence of macroscopically observable fragmentation and fissuring in an area 1.27 cm or less in diameter. Grade III represents fragmentation and fissuring that occupy an area more than 1.27 cm in diameter. Grade IV is characterized by erosion of cartilage with eburnation [1]. The meniscal condition was also recorded. A complete meniscal tear or defect involving more than half of the meniscus, including meniscectomy status, was defined as high-grade meniscus damage. The mean grade from three evaluators was recorded both at ACL reconstruction (baseline) and implant removal (follow-up). Inter-observer error was found to be 9.1% [1].

### Clinical evaluation of ACL reconstruction

Results of ACL reconstruction were evaluated by radiographic examination, using the Tegner activity level scale and the pivot shift test. Knee radiographs were examined and graded according to the Kellgren-Lawrence scale [12]. Higher than grade I was defined as radiographic OA. The Tegner activity level scale provides numerical grades for work and sport activities and assesses disability after knee ligament injury [13]. The pivot shift test assesses knee stability, particularly with regard to rotatory instability after reconstruction [14].

### Sampling of SF and biomarker measurements

Sampling was performed at baseline, the time of ACL reconstruction prior to arthroscopic evaluation using a lateral suprapatellar approach. Samples were centrifuged at 10,000 rpm for 20 min at 4 °C and the cell-free supernatants were stored at -80 °C until analyzed [1]. C2C, KS, Δdi-C4S, and Δdi-C6S levels in SF samples were measured by ELISA (C2C) or high-performance liquid chromatography (KS, Δdi-C4S, and Δdi-C6S), as previously described [1]. Moreover, in consideration of our previous finding that the ratio of C2C to KS (C2C/KS) may identify patients with early cartilage damage [1], or the reports that the ratio of Δdi-C6S to Δdi-C4S (C6S/C4S) was associated with age, sex, and the progression of OA [15, 16], we added C2C/KS and C6S/C4S to the analyses.

### Progression of cartilage damage

We defined grade III and IV lesions as high-grade cartilage damage [1]. Grade III encompasses a relatively wide range of cartilage damage and is easily distinguishable from grade II. Changes in the number of high-grade lesions (grades III and IV) from the first examination (baseline) to the second examination (follow-up) at the six articular sites were measured. The increased number of high-grade lesions (grades III and IV) indicated that the cartilage damage had increased and was approaching that observed in radiographic OA. Hence, progression of cartilage damage was defined as an increased number of high-grade cartilage lesions (grades III and IV). Of the 62 patients who were followed in this study, 15 exhibited progression of cartilage damage (progression group).

### Statistical analysis

All statistical analyses were performed with EZR (Saitama Medical Center, Jichi Medical University, Saitama, Japan), a graphical user interface for R (The R Foundation for Statistical Computing, Vienna, Austria) [17]. *P* < 0.05 was considered statistically significant.

Student’s *t* test was used to analyze continuous variables. Fisher’s exact test was used to analyze ordinal variables and categorical variables. When comparing levels of biomarkers between progression and non-progression groups, the non-parametric Mann-Whitney *U* test was used.

Receiver operating characteristic (ROC) curves were generated to assess associations between levels of biomarkers and progression of high-grade cartilage damage. The best cut-off point was identified as the maximum point of the Youden index, which was calculated using the following formula: Youden index = sensitivity + specificity - 1.

Finally, multivariable logistic regression analyses were performed to confirm the independent impact of variables on the progression of high-grade cartilage damage. In order to dichotomize values, cut-off points based on ROC curves were used.

## Results

### Lesion onset and progression

Table 1 summarizes the characteristics of patients by group (progression group, n = 15; non-progression group, n = 47). The mean age was higher in the progression group (31.1 years) than in the non-progression group (26.5 years). The mean duration from injury to ACL reconstruction was longer in the progression group than in the non-progression group. There were almost no differences between groups in terms of proportion of females, proportion of high-grade meniscus damage, or proportion with more than one grade III lesion.

**Table 1:** Characteristics of patients in the progression group and the non-progression group at baseline and follow-up

Variables		Total (n = 62)	Progression group (n = 15)	Non-progression group (n = 47)	*p* value
Baseline					
Age (years)	Mean (SD)	27.6 (9.0)	31.1 (9.2)	26.5 (8.7)	0.083
Duration from injury to ACL reconstruction (months)	Mean (SD)	26.1 (37.8)	39.7 (49.8)	21.8 (32.6)	0.111
Sex, female (%)		38.7	40	38.3	1
High-grade meniscus damage (%)		58.1	66.7	55.3	0.553
More than one grade III or IV lesion (%)		35.5	46.7	31.9	0.359
Follow-up					
More than grade I of Kellgren-Lawrence (%)		0	0	0	1
Duration from reconstruction to removal (months)	Mean (SD)	26.0 (7.8)	27.3 (11.7)	25.6 (6.2)	0.482
BMI	Mean (SD)	24.1 (3.1)	23.9 (2.9)	24.2 (3.2)	0.744
Tegner activity level scale	Mean (SD)	5.1 (2.2)	5.1 (2.5)	5.1 (2.1)	0.944
Pivot shift test, positive (%)		18.3	6.7	22.2	0.262
High-grade meniscus damage (%)		64.5	60.0	68.1	0.755
More than one grade III or IV lesion (%)		40.3	100	21.3	<0.001

Baseline, ACL reconstruction; Follow-up, implant removal; Progression group, patients with an increase (between baseline and follow-up) in the number of high-grade cartilage lesions (Outerbridge grades III and IV) by one score in any of the six compartments; Non-progression group, patients with no increase in the number of high-grade cartilage lesions; *BMI* body mass index; high-grade meniscus damage, complete meniscal tear or defect in more than half the meniscus; *SD* standard deviation. *P* < 0.05 was considered statistically significant

Patient characteristics at follow-up are also shown in Table 1. There were no patients with radiographic OA (more than grade I Kellgren-Lawrence) that showed joint space narrowing, osteophytes, and subchondral and sclerotic bone lesions. There were almost no differences between progression and non-progression groups in the duration from first to second evaluation, BMI, Tegner activity level scale, proportion of those with a positive pivot shift test, and proportion of those with high-grade meniscus damage. The proportion of those with more than one Grade III lesion at the second evaluation was higher in the progression group (100%) than in the non-progression group (21.3%).

Figure 1 shows the proportions of high-grade cartilage damage in the progression and non-progression groups at baseline and follow-up. At follow-up, more than half of those in the progression group had high-grade damage at the femoral grove and lateral tibial plateau.

Table 2 shows changes in the number of grade 0–IV lesions from baseline to follow-up. In both groups, the number of grade I lesions was higher at baseline. In the progression group, the numbers of grade 0 (normal cartilage), grade I, and grade II lesions that progressed to high-grade lesions were 4, 6, and 10, respectively: not only well-established lesions but also normal cartilage and lesions showing minimal damage exhibited development and progression of cartilage damage, respectively, based on arthroscopic evaluation. In contrast, in the non-progression group, almost all low-grade lesions remained unchanged, while some of high-grade lesions changed to low-grade lesions (n = 11).

**Table 2:** Number of grade 0–IV lesions from baseline to follow-up in the progression and the non-progression groups

Progression group					Non-progression group				
Baseline			Follow-up		Baseline			Follow-up	
			Grade 0–II	Grade III–IV				Grade 0–II	Grade III–IV
Grade 0	28.9%	(n = 26)	22 (84.6%)	4 (15.4%)	Grade 0	28.7%	(n = 81)	81 (100%)	0 (0%)
Grade I	33.3%	(n = 30)	24 (80.0%)	6 (20.0%)	Grade I	44.3%	(n = 125)	122 (97.6%)	3 (2.4%)
Grade II	26.7%	(n = 24)	14 (58.3%)	10 (41.7%)	Grade II	17.7%	(n = 50)	49 (98.0%)	1 (2.0%)
Grade III–IV	11.1%	(n = 10)	0 (0%)	10 (100%)	Grade III–IV	9.2%	(n = 26)	11 (42.3%)	15 (57.7%)

N = 15 (progression group), N = 47 (non-progression group). Total compartments that were investigated arthroscopically for the Outerbridge grading = 6 per knee (total of 90 (6 × 15) compartments for the progression group and 282 (6 × 47) compartments for the non-progression group). Baseline, ACL construction; Follow-up, implant removal; Progression group, patients with an increase (between baseline and follow-up) in the number of high-grade cartilage lesions (Outerbridge grades III and IV) by one score in any of the six compartments; Non-progression group, patients with no increase in the number of high-grade cartilage lesions

### Biomarker evaluation

With regard to biomarkers of SF at the time of ACL reconstruction (baseline), levels of Δdi-C6S and KS, as well as the ratio of Δdi-C6S to Δdi-C4S (C6S/C4S), were significantly lower in the progression group compared to the non-progression group. There were no differences in levels of C2C, Δdi-C4S and the ratio of C2C to KS (C2C/KS) between the two groups (Table 3).

**Table 3:** Biomarker levels of patients in the progression and non-progression groups at ACL reconstruction (baseline)

Variables		Total (n = 62)	Progression group (n = 15)	Non-progression group (n = 47)	*p* value
C2C (ng/ml)	Median (IQR)	9.8 (5.8–13.8)	8.8 (5.6–11.8)	10.4 (6.1–14.3)	0.282
Δdi-C6S (nmol/ml)	Median (IQR)	69.6 (54.9–95.1)	53.4 (50.9–69.4)	73.5 (60.7–99.9)	0.004
Δdi-C4S (nmol/ml)	Median (IQR)	17.1 (14.2–21.6)	16.3 (14.8–17.8)	18.0 (14.2–23.0)	0.170
KS (μg/ml)	Median (IQR)	11.6 (9.4–15.6)	9.9 (8.3–11.4)	11.9 (10.0–16.0)	0.021
C2C/KS ratio	Median (IQR)	0.71 (0.51–1.2)	0.67 (0.52–1.2)	0.75 (0.51–1.2)	0.928
C6S/C4S ratio	Median (IQR)	4.2 (3.6–4.9)	3.9 (3.2–4.4)	4.4 (3.7–5.2)	0.028

Progression group, patients with an increase (between baseline and follow-up) in the number of high-grade cartilage lesions (Outerbridge grades III and IV) by one score in any of the six compartments; Non-progression group, patients with no increase in the number of high-grade cartilage lesions; *IQR* interquartile range. *P* < 0.05 was considered statistically significant

**Fig. 2:**
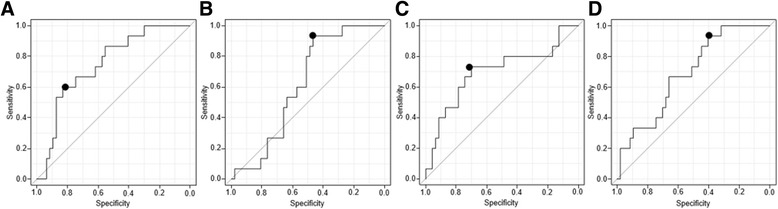
ROC curves for Δdi-C6S level, Δdi-C4S level, KS level, C6S/C4S, and the presence of high-grade cartilage progression. *Black dots* in the figure panels indicate cut-off points. **a** ROC analysis for the level of Δdi-C6S and the presence of high-grade cartilage progression (area under the ROC curve: 0.746 [95% CI 0.612–0.880]; 60.0% sensitivity and 83.0% specificity for a cut-off of 55.7 nmol/ml). **b** ROC analysis for the level of Δdi-C4S and the presence of high-grade cartilage progression (area under the ROC curve 0.618 [95% CI 0.478–0.758]; 93.3% sensitivity and 46.8% specificity for a cut-off of 19.0 nmol/ml). **c** ROC analysis for the level of KS and the presence of high-grade cartilage progression (area under the ROC curve: 0.699 [95% CI 0.526–0.872]; 73.3% sensitivity and 70.2% specificity for a cut-off of 10.6 μg/ml). **d** ROC analysis for C6S/C4S (ratio of Δdi-C6S to Δdi-C4S) and the presence of high-grade cartilage progression (area under the ROC curve: 0.689 [95% CI 0.546–0.833]; 93.3% sensitivity and 40.0% specificity for a cut-off of 4.6)

Using ROC analysis, we assessed the associations between the progression of high-grade cartilage damage and baseline levels of Δdi-C6S, Δdi-C4S and KS as well as the C6S/C4S ratio (Fig. 2). The areas under the ROC curve were 0.746 (95% CI 0.612–0.880) for Δdi-C6S, 0.618 (95% CI 0.478–0.758) for Δdi-C4S, 0.699 (95% CI 0.526–0.872) for KS, and 0.689 (95% CI 0.546–0.833) for C6S/C4S. Best cut-off values were 55.7 nmol/ml for Δdi-C6S (sensitivity 60.0%, specificity 83.0%), 19.0 nmol/ml for Δdi-C4S (sensitivity 93.3%, specificity 46.8%). 10.6 μg/ml for KS (sensitivity 73.3%, specificity 70.2%), and 4.6 for C6S/C4S (sensitivity 93.3%, specificity 40.0%).

Table 4 shows the associations of Δdi-C6S levels, Δdi-C4S levels, KS levels, and the C6S/C4S ratio with the progression of high-grade cartilage damage as revealed by logistic regression analyses. Even after adjusting for age, the duration from injury to ACL reconstruction, sex, and the number of high-grade lesions (grades III and IV) at baseline, associations remained significant between the progression of high-grade cartilage damage and levels of Δdi-C6S (OR 0.231, 95% CI 0.061–0.879), levels of Δdi-C4S (OR 0.081, 95% CI 0.008–0.763), KS (OR 0.114, 95% CI 0.024–0.529), and the ratio of C6S/C4S (OR 0.060, 95% CI 0.005–0.737).

**Table 4:** Odds ratios for the presence of high-grade cartilage damage progression by Δdi-C6S, KS levels, C6S/C4S

Variables		Unadjusted OR			Adjusted OR		
			(95% CI)	*p* value		(95% CI)	*p* value
Δdi-C6S	≤55.7	1	Reference		1	Reference	
(nmol/ml)	>55.7	0.179	(0.051–0.638)	0.008	0.231	(0.061–0.879)	0.032
Δdi-C4S	≤19.0	1	Reference		1	Reference	
(nmol/ml)	>19.0	0.081	(0.010–0.668)	0.020	0.081	(0.008–0.763)	0.028
KS	≤10.6	1	Reference		1	Reference	
(μg/ml)	>10.6	0.154	(0.042–0.568)	0.005	0.114	(0.024–0.529)	0.006
C6S/C4S	≤4.6	1	Reference		1	Reference	
	>4.6	0.105	(0.013–0.869)	0.037	0.060	(0.005–0.737)	0.028

All independent variables were dichotomized using the cut-off points calculated by ROC analysis and tested in a binary manner. The adjusted model included variables (age, duration from injury to ACL reconstruction, sex, and the number of high-grade cartilage lesions [Outerbridge grades III and IV]) at baseline. *P* < 0.05 was considered statistically significant

## Discussion

We found that SF levels of Δdi-C6S, Δdi-C4S, KS and the ratio of Δdi-C6S to Δdi-C4S (C6S/C4S) are significantly associated with the onset and progression of pre-radiographic high-grade focal cartilage damage—which was not reflected by a transition to detectable radiographic changes—based on longitudinal arthroscopic and radiographic evaluations. Many studies have reported on biomarkers associated with cartilage damage progression with regard to radiographic changes [18–23]. This study is the first to report that biomarkers of SF can predict pre-radiographic onset and progression of early cartilage damage on the basis of longitudinal arthroscopic observations.

In both the progression and non-progression groups, arthroscopic evaluations of morphological cartilage damage at baseline revealed similar numbers of grade 0–IV lesions (Table 2). In fact, morphologically normal cartilage and minimally damaged lesions (grade 0–I) exhibited onset and progression of cartilage damage, respectively, in the progression group (Table 2). Interestingly, cut-off values of 55.7 nmol/ml for Δdi-C6S, 10.6 μg/ml for KS, and 4.6 for C6S/C4S, as determined by ROC analysis (Fig. 2), correspond to grades 2–4 according to the Kellgren-Lawrence grading scale [6, 10, 24], or advanced OA [25]. This suggests that those showing progression of high-grade cartilage damage exhibited a cartilage aggrecan metabolism similar to that observed in patients with advanced radiographic OA [12], although no radiographic OA changes were observed in this study. Therefore, cut-off values of Δdi-C6S, KS, and C6S/C4S could be used as biomarkers to predict onset/progression of cartilage damage. Changes in proteoglycans, almost certainly released primarily from aggrecan because of its much greater content of these glycosaminoglycans, reflect cartilage metabolism at an early stage of OA [26]. Although the loss of aggrecan leads to increased aggrecan synthesis, newly synthesized molecules are composed of enriched C4S instead of C6S and KS [8–10], unlike those found in normal SF [16]. Our results are consistent with previous reports [8–10, 16], in that levels of Δdi-C6S and KS and the ratio of C6S/C4S were negatively correlated with cartilage damage progression.

With regard to Δdi-C4S, when this was added to the logistic regression analysis, a significant difference was noted such that progression of cartilage damage did not advance if Δdi-C4S was above 19.0 nmol/ml (calculated from the ROC curve) (Table 4), although a significant difference was not found between the progression group and non-progression group (Table 3), and the area under the ROC curve for Δdi-C4S was 0.618, which was lower than that for Δdi-C6S (0.746), KS (0.699), and C6S/C4S (0.689) (Fig. 2). Normally, Δdi-C4S is highly expressed in OA cartilage [8–10], which seems to contradict the result of this study. However, Δdi-C4S and Δdi-C6S showed a significant positive correlation (correlation coefficient = 0.838 [95% CI 0.744–0.899, *p* = 0]) with Pearson’s product-moment correlation in this study. We surmise that, with regard to early changes following trauma, as long as synthesis of Δdi-C6S was greater than that of Δdi-C4S, progression of cartilage damage could not advance. Therefore, it is appropriate to consider also the ratio of C6S/C4S as a predictive biomarker for progression of cartilage damage.

Our previous cross-sectional study found that C2C, which reflects increased collagenase cleavage of cartilage type II collagen, was significantly associated with the presence of arthroscopic high-grade cartilage damage [1]. C2C is considered a sensitive marker for detecting early cartilage damage. However, in the present longitudinal study, C2C was not associated with the onset/progression of high-grade cartilage damage. Mean SF C2C level in OA patients (n = 54) was reported to be 30.5 ng/ml in a previous study [27], whereas the median C2C level in the present study was 9.8 ng/ml (interquartile range, 5.8–13.8). Since our patients were at the pre-radiographic OA stage, their C2C levels might have been lower than those of OA patients and thus did not predict the progression of cartilage damage. In a recent study, a C2C-HUSA urine assay, which measures a more specific degradation product(s), revealed the association between baseline data and knee OA progression [18].

Older patients reportedly have increased cartilage damage and more severe OA changes after ACL injury [28]. Age is negatively correlated with the ratio of C6S/C4S [15], and the C6S/C4S ratio is typically lower in females than in males [15]. This is likely due to the decreased cartilage repair capacity in older patients, and may even reflect gender specificity. In a previous study, we found that both age and duration from injury to first surgery were positively correlated with the number of high-grade cartilage lesions [1]. Moreover, mean age and mean duration were higher in the progression group compared to the non-progression group (Table 1). In addition, baseline cartilage damage might be an important driver for cartilage damage progression. Thus, age, duration from injury to first surgery, sex, and the number of high-grade lesions (grades III and IV) at baseline should all be taken into account when predicting cartilage damage. As shown in Table 4, the levels of Δdi-C6S and KS and the ratio of C6S/C4S were associated with the progression of high-grade cartilage damage, even after adjusting for age, duration from injury to first surgery, sex, and the number of high-grade lesions (grades III and IV) at baseline in multivariable logistic regression analyses. Therefore, levels of Δdi-C6S and KS and the C6S/C4S ratio could serve as meaningful predictive biomarkers.

Meniscal tears or defects are risk factors for knee OA [29]. However, the proportion of high-grade meniscus damage in the progression group was similar to that in the non-progression group, at both the first and second evaluations (Table 1). The main component of the meniscus is type I collagen. In contrast to articular cartilage that mainly comprises type II collagen and contains abundant proteoglycans, the meniscus is highly deficient in the proteoglycans [30]. In this regard, meniscus damage is unlikely to contribute to differences in the biomarkers in both groups.

Whether ACL reconstruction performed at our institute is reproducible or not is of considerable importance. All ACL reconstructions were performed by three senior surgeons who had operated on more than 100 cases. Clinical results of ACL reconstruction and arthroscopic longitudinal changes of cartilage damage after ACL reconstruction in this study were comparable to those of previous reports [31, 32].

There are some limitations to this study. First, only a limited number of biomarkers were evaluated. Various biomarkers that reflect cartilage metabolism are available [33], including procollagen II C-propeptide (CPII) [34], which serves as an indicator of collagen synthesis. Indeed, the progression of OA might reflect collagen synthesis rather than collagen cleavage [35]. Thus, changes in collagen metabolism in the early stages of OA should be explored. Second, as this study was conducted retrospectively, the duration from the first to second evaluation varied by patient. That said, the duration was approximately 2 years in most patients in both groups, and given the lack of significant differences between the two groups, its influence was likely minimal. Third, the sample size is relatively small for evaluation of predictive biomarkers. Thus, a bigger group will be needed to demonstrate whether the obtained cut-off values are proper for prediction of cartilage damage progression. Finally, the present study examined cartilage damage after ACL injury based on longitudinal arthroscopic observation, but did not address whether cartilage damage will progress further to radiographic OA damage. A longer observation period would be required to address this issue.

## Conclusions

Progression of high-grade cartilage damage as evaluated by arthroscopic evaluation was significantly associated with biomarkers of aggrecan glycosaminoglycan metabolism; namely, levels of Δdi-C6S and KS and the ratio of Δdi-C6S to Δdi-C4S. The cut-off values determined in this study may be useful for predicting the progression of cartilage damage.

## Additional files

Additional file 1: Supplementary data for patient baseline characteristics of 62 patients in the current study and 46 patients who dropped out of our follow-up study. (XLS 30 kb)

Additional file 2: Datasets generated during the current study. (CSV 11 kb)

## Abbreviations

ACL: Anterior cruciate ligament
C2C: Collagenase-generated cleavage neoepitope of type II collagen
KS: Keratan sulfate
OA: Osteoarthritis
SF: Synovial fluid
Δdi-C4S: Chondroitin-4-sulfate
Δdi-C6S: Chondroitin-6-sulfate
